# Retraction Note: Mothers’ experiences of caring for preterm babies at home: qualitative insights from an urban setting in a middle-income country

**DOI:** 10.1186/s12884-021-04185-7

**Published:** 2021-10-19

**Authors:** Isabella Garti, Elorm Donkor, Nafisatu Musah, Evans Osei Appiah, Sandra Gyekye, Awube Menlah, Cynthia Pomaa Akuoko

**Affiliations:** 1 College of Nursing and Midwifery, Charles Darwin University, Darwin, Australia; 2 Department of Nursing and Midwifery, Valley View University, Accra, Ghana; 3 Department of Nursing and Midwifery, Greater Accra Regional Directorate, Ghana Health Service, Accra, Ghana; 4 Department of Nursing and Midwifery, Trust Mother and Child Hospital, Accra, Ghana; 5 Department of Nursing, Christian Service University College, Kumasi, Ghana; 6 School of Nursing, Queensland University of Technology, Brisbane, Australia

**Retraction Note: BMC Pregnancy Childbirth 21, 395 (2021)**

**https://doi.org/10.1186/s12884-021-03872-9**

The authors have retracted this article. After publication they found that the data are unreliable and they no longer have confidence in the results and conclusions presented. All authors agree with this retraction.

